# Cake Layer Fouling Potential Characterization for Wastewater Reverse Osmosis via Gradient Filtration

**DOI:** 10.3390/membranes12080810

**Published:** 2022-08-21

**Authors:** Rulu Ouyang, Bin Huang, Chun-Hai Wei, Hongwei Rong, Huarong Yu, Fangshu Qu, Kang Xiao, Xia Huang

**Affiliations:** 1 Department of Municipal Engineering, School of Civil Engineering, Guangzhou University, Guangzhou 510006, China; 2 China Railway Conservancy & Hydropower Planning and Design Group Co., Ltd., Nanchang 330029, China; 3 Key Laboratory for Water Quality and Conservation of the Pearl River Delta, Ministry of Education, Guangzhou 510006, China; 4 College of Resources and Environment, University of Chinese Academy of Sciences, Beijing 101408, China; 5 State Key Joint Laboratory of Environmental Simulation and Pollution Control, School of Environment, Tsinghua University, Beijing 100084, China

**Keywords:** gradient filtration, single and combined membrane fouling models, cake layer fouling index, fouling potential, model foulants, real wastewater, reverse osmosis

## Abstract

It is of great importance to quantitatively characterize feed fouling potential for the effective and efficient prevention and control of reverse osmosis membrane fouling. A gradient filtration method with microfiltration (MF 0.45 μm) → ultrafiltration (UF 100 kDa) → nanofiltration (NF 300 Da) was proposed to extract the cake layer fouling index, *I*, of different feed foulants in this study. MF, UF, and NF showed high rejection of model suspended solids (kaolin), colloids (sodium alginate and bovine serum albumin), and dissolved organic matters (humic acid) during constant-pressure individual filtration tests, where the cake layer was the dominant fouling mechanism, with *I* showing a good linear positive correlation with the foulant concentration. MF → UF → NF gradient filtration tests of synthetic wastewater (i.e., model mixture) showed that combined models were more effective than single models to analyze membrane fouling mechanisms. For each membrane of gradient filtration, *I* showed a positive correlation with the targeted foulant concentration. Therefore, a quantitative assessment method based on MF → UF → NF gradient filtration, the correlation of combined fouling models, and the calculation of *I* would be useful for characterizing the fouling potentials of different foulants. This method was further successfully applied for characterizing the fouling potential of real wastewater (i.e., sludge supernatant from a membrane bioreactor treating dyeing and finishing wastewater).

## 1. Introduction

A reverse osmosis (RO) membrane with a nonporous dense structure has the highest rejection performance (theoretically rejects any substance except for water molecules) among pressure-driven membranes for water and wastewater treatment [1]. Thus, the RO process has gained successful full-scale application in seawater desalination [2,3,4,5] and high-quality reclaimed water production, mainly from biologically treated wastewater [6,7,8], due to its intrinsic advantages of stable and excellent permeate quality, compact structure and small footprint, ease of automatic operation, etc. However, membrane fouling is an inevitable problem during RO membrane operation, resulting in a reduction in membrane flux and rejection performance, thus restricting its technical economy [9,10,11]. Membrane fouling is essentially affected by the complex interactions between the membrane and foulants and among different foulants [12,13,14]. It is of great importance to accurately and quickly characterize the fouling potential of RO feed to select an appropriate pretreatment to effectively and efficiently prevent and control RO membrane fouling [15,16]. Thus, a fouling index is proposed to quantitatively characterize the fouling potential of RO feed [17,18,19].

The silt density index (SDI) was first developed to predict the fouling potential of particles (including suspended solids and some colloids) in RO feed [20]. It measures the plugging rate of a microfiltration (MF) membrane (mean pore size of 0.45 µm and diameter of 47 mm) in a constant-pressure (207 kPa) dead-end filtration mode. Lots of statistical data have demonstrated the significant positive correlation between RO membrane fouling and the SDI of RO feed [20]. The SDI of RO feed has been listed as one of the key feed quality parameters by RO membrane manufactures. Based on field experience, the SDI of RO feed is generally required to be less than 4 or 5 for a spiral-wound module and less than 3 for a hollow-fiber module. However, the SDI is inherently an empirical parameter characterizing the MF fouling rate in constant-pressure dead-end filtration and thus lacks a theoretical basis. Many studies have shown the reliability issue of SDI testing and the limitations of SDI application (e.g., the lack of a fouling mechanism, no intrinsic linear relationship between the SDI value and the concentration of colloid and suspended solids, and not covering most colloids with sizes significantly less than 0.45 µm) [21,22,23].

The modified fouling index (MFI) was derived directly from the improvement of the SDI, where the same MF process and a new calculation method based on a cake layer fouling mechanism were adopted [24]. Thus, the MFI is better than the SDI to characterize the particulate fouling potential of RO feed. Some RO membrane manufacturers have required feed MFI less than 1 s/L^2^, which is equivalent to an SDI less than 3 according to the standard method for MFI measurement [24]. As a relatively new index, the MFI still needs time to accumulate field data for setting the target value of RO feed. In addition, pore blocking and cake layer fouling mechanisms usually occur simultaneously during the constant-pressure dead-end MF process [24], thus affecting MFI measurement. A new double-MFI measurement (i.e., raw feed for the first measurement and the first permeate for the second measurement) was proposed to eliminate the effects of pore blocking [25].

Considering that SDI and MFI measurements are based on an MF membrane with a mean pore diameter of 0.45 μm that cannot reject colloids and dissolved organic matters (i.e., the important foulants causing RO fouling) effectively, an ultrafiltration (UF) membrane with a molecular weight cut-off (MWCO) of 13 kDa (approximate pore size of 5–8 nm) and a nanofiltration (NF) membrane with an MWCO of 1.5 kDa were proposed for MFI measurements, resulting in MFI-UF [26] and MFI-NF [27]. MFI-UF has been developed under both constant-pressure and constant-flux dead-end filtration modes [28,29]. MFI-UF with a crossflow sampler has been further developed to simulate the hydraulic selective deposition of particulates and colloids during real crossflow filtration of an RO membrane [30]. MFI-NF with a cake resistance simulator has been further proposed to simulate the same salt concentration close to the RO membrane surface and filtration pressure as the real RO process, resulting in the same specific cake resistance [31]. It should be noted that the last two improvements require a complex measuring device and methodology, take a long time, and need more field data verification.

There are generally multiple potential foulants with sizes of several nanometers to several hundred micrometers (i.e., particulates, colloids, and dissolved organic matters) in RO feed (e.g., seawater and biologically treated wastewater), resulting in very complex fouling phenomena, and thus it is very difficult to characterize its fouling potential via only one index associated with one membrane. Thus, a multiple membrane array system with gradient filtration of MF → UF → NF was proposed to characterize the cake layer fouling potentials of particulate matters, colloids, and dissolved organic matters via MFI-MF, MFI-UF, and MFI-NF, respectively, for a seawater RO application [32,33]. Although it is a promising methodology to characterize the fouling potential of RO feed, the gradient filtration of MF → UF → NF requires further development, including membrane and pressure selection, the extraction methodology of the real cake layer fouling potential, and verification from seawater as well as biologically treated wastewater.

In this study, an MF → UF → NF gradient filtration methodology was systematically investigated to characterize the fouling potential of biologically treated wastewater as a targeted RO feed. The positive correlations between the cake layer fouling index and the targeted foulant concentrations were first demonstrated for individual MF, UF, and NF tests. Then, single and combined membrane fouling models were employed to analyze the fouling mechanism of each membrane (MF, UF, and NF) filtration process in the MF → UF → NF gradient filtration of a model foulant mixture, resulting in a new extraction method based on combined models for the cake layer fouling index. Thus, the MF → UF → NF gradient filtration methodology was proposed because the cake layer fouling index showed a positive correlation with feed quality for each membrane. Finally, the MF → UF → NF gradient filtration methodology was successfully employed to characterize the fouling potential of biologically treated dyeing and finishing wastewater.

## 2. Materials and Methods

### 2.1. Experimental Setup for Gradient Filtration

**Figure 1:**
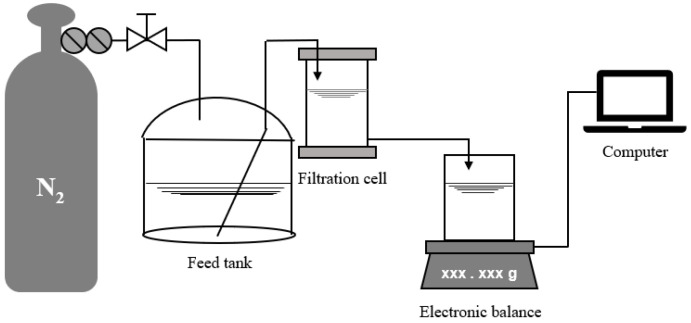
Low-pressure MF/UF dead-end filtration setup.

**Figure 2:**
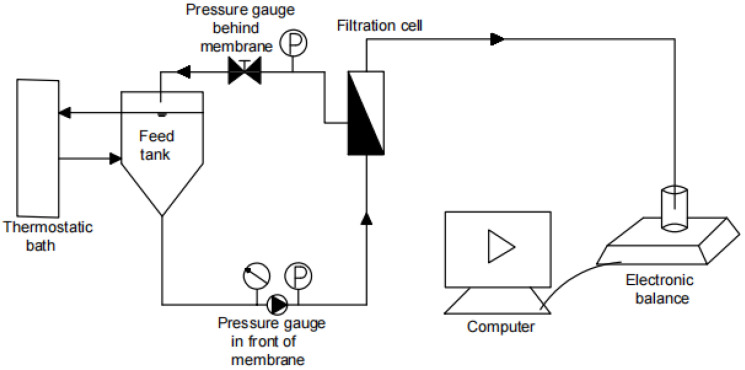
High-pressure NF crossflow filtration setup.

In this study, a lab-scale low-pressure MF/UF dead-end filtration setup (shown in Figure 1) and a high-pressure NF cross-flow filtration setup (shown in Figure 2) were used for gradient filtration tests. Through pretests, a polyvinylidene fluoride flat-sheet MF membrane (model name of MF045) with a nominal pore size of 0.45 μm, a polyvinylidene fluoride flat-sheet UF membrane (model name of UF100) with an MWCO of 100 kDa, and a composite polyamide flat-sheet NF membrane (model name of NF200) with an MWCO of 300 Da (MF and UF from RisingSun Membrane Technology, Beijing, China and NF from Dow Chemical, Midland, MI, USA) were selected for the gradient filtration tests of MF → UF → NF, where the corresponding operating pressures were selected as low values of 0.5, 1, and 10 bar, respectively, considering the potential occurrence of cake layer compression and balance recording malfunction under high pressure and the associated high initial permeate flow. All membrane surfaces were hydrophilic, and the pure water fluxes at 20 °C of the MF, UF, and NF membranes were measured as 7200, 168, and 3.1 L/m^2^∙h/bar, respectively, in this study. The surface mean roughness of the NF membrane was reported as 5.2 nm in a previous study [34].

Prior to the beginning of the low-pressure MF/UF dead-end filtration test, compressed nitrogen was first employed to pressurize the water sample to be filtered in a stainless-steel tank with an effective volume of 10 L to a preset pressure value. Then, the exhaust valve of the filtration cell with a membrane area of 41.8 cm^2^ (Amicon 8400, Millipore, Burlington, MA, USA) was opened to vent the residual air in the pipe and filtration cell. The constant-pressure dead-end filtration was thus started. The weight of the filtrate was weighed in real time by an electronic balance (ME4002E, Mettler Toledo, Greifensee, Switzerland) and sent to a computer for recording. According to the measured temperature of the filtrate (20 ± 1 °C), the density was calculated, and the weight was further converted into volume. Finally, the instantaneous filtration rate was obtained by the numerical differentiation of the filtrate volume and the filtration time, and the instantaneous membrane flux was further calculated.

The NF cross-flow filtration setup was composed of a high-pressure plunger diaphragm pump, a flat-sheet NF membrane with an area of 96 cm^2^, an inlet/concentrate bucket, a thermostatic bath, two pressure gauges, a regulating valve, a balance for measuring effluent weight, and a computer. The high-pressure pump provided a constant-pressure cross-flow filtration mode. The pressure was adjusted by the regulating valve. The thermostatic bath maintained the water sample at a constant temperature of 20 ± 1 °C through a heat exchange jacket. The filtrate was automatically weighed and recorded by the balance connected to the computer and further processed according to the above-mentioned method.

### 2.2. Wastewater Sample and Analytical Method

The model foulants of kaolin (mean diameter of 2.5 μm), a mixture of sodium alginate (mean MWCO of 150 kDa) and bovine serum albumin (mean MWCO of 66 kDa), and humic acid (mean MWCO of 3 kDa) from Macklin (Shanghai, China) were employed to simulate suspended solids, colloids, and dissolved organic matters in biologically treated wastewater. The synthetic kaolin solution (10, 50, and 100 mg/L), the mixed solution of sodium alginate (10, 50, and 100 mg/L) and bovine serum albumin (10, 50, and 100 mg/L), and the humic acid solution (10, 50, and 100 mg/L), fully mixed or dissolved by stirring, were prepared for the individual MF, UF, and NF tests, respectively. The synthetic wastewater (SWW) sample for the gradient filtration test of MF → UF → NF was a mixture of the model foulants. High- and low-concentration samples containing kaolin (10 and 5 mg/L), sodium alginate (1 and 0.5 mg/L), bovine serum albumin (1.5 and 0.75 mg/L), and humic acid (7.5 and 3.75 mg/L), respectively, were prepared to simulate high- and low-concentration biologically treated wastewater as SWW samples. The sludge supernatant from a membrane bioreactor (MBR) treating dyeing and finishing wastewater in a local wastewater treatment plant was prepared via gravitational sedimentation as a real wastewater (RWW) sample.

The turbidity and absorbance at 254 nm (UV_254_) of the wastewater samples were measured by a portable turbidimeter (WGZ-4000B, Xinrui, Shanghai, China) and an ultraviolet-visible spectrophotometer (T6, Puxi, Beijing, China), respectively. The raw wastewater samples containing suspended solids were prefiltered by a 0.45 μm syringe filter to measure dissolved organic carbon (DOC) with an organic carbon analyzer (TOC-L, Shimadzu, Kyoto, Japan). The DOC values of membrane permeate samples were directly measured.

### 2.3. Membrane Fouling Mechanism Analysis

Table 1 lists the single and combined membrane fouling models that can be used to describe the constant-pressure filtration process [23,35,36]. In this study, Origin software (2018, OriginLab, Northampton, MA, USA) was used to fit the filtration data with the model equations for analyzing the membrane fouling mechanism. The linear and nonlinear fitting for single and combined membrane fouling models, respectively, were conducted based on the equations in Table 1. On the basis of passing the model *F* test and the parameter *t* test (*p* < 0.1), a coefficient of determination, R^2^, of more than 0.95 could be considered as a successful fitting.

Based on the cake layer fouling model in Table 1, the MFI is calculated from Equation (1) to evaluate the fouling potential of the cake layer [24].

(1) $$\frac{t}{V}=\frac{1}{AJ_{0}}+\frac{K_{c}}{2A^{2}}V=\frac{\mu R_{m}}{A\Delta P}+\frac{\mu I}{2\Delta PA^{2}}V=\frac{\mu R_{m}}{A\Delta P}+\frac{\mu \alpha C}{2\Delta PA^{2}}V=\frac{\mu R_{m}}{A\Delta P}+\mathrm{MFI}\cdot V$$

where Δ*P* is the filtration pressure (N/m^2^), *μ* is the filtrate viscosity (N∙s/m^2^), *R_m_* is the pure membrane resistance (m^−1^), *I* is the fouling index of the cake layer (1/m^2^), *α* is the specific resistance of the cake layer (m/g), *C* is the particulate matter concentration (mg/L), and MFI is the modified fouling index (s/L^2^). *K_c_* can be obtained by linear fitting, and the MFI and *I* can be further calculated according to *A*, Δ*P*, and *μ*. In this study, the MFI values for the filtration tests of MF, UF, and NF were not directly comparable due to the different pressure and membrane area applied, while *I* was the product of *α* and *C* and thus directly characterized the cake layer formed on the membrane surface. Therefore, the cake layer fouling index, *I*, of MF, UF, and NF in MF → UF → NF gradient filtration could be used to quantitatively compare the cake layer fouling potential of suspended solids, colloids, and dissolved organic matters, respectively.

## 3. Results and Discussion

### 3.1. Response of Cake Layer Fouling Index, I, to Targeted Foulant Concentrations for Individual MF, UF, and NF Tests

**Figure 3:**
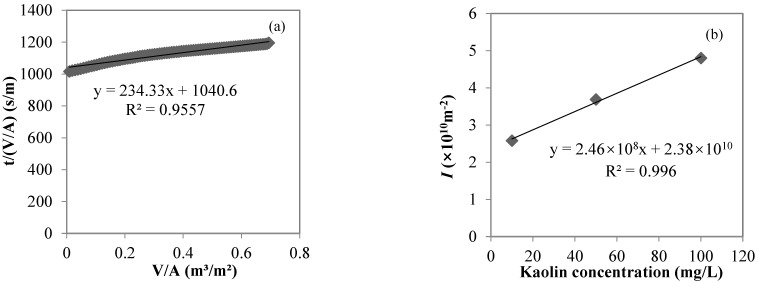
Cake layer model fitting for the MF process of a 10 mg/L kaolin solution (**a**) and the correlation between *I* and the feed kaolin concentration (**b**).

The turbidities of the kaolin solutions with concentrations of 10, 50, and 100 mg/L were 0.83, 2.86, and 5.48 NTU, respectively; the turbidities of the MF permeates were 0.32, 0.40, and 0.61 NTU, respectively; and the turbidity rejection rates of MF were 62.8, 86.0, and 90.0%, respectively, indicating that the MF membrane with a nominal pore size of 0.45 μm used in the study could effectively reject suspended solids, represented by kaolin. The MF process closely followed the cake layer model in this study. Taking the filtration of a 10 mg/L kaolin solution as an example, the cake layer model fit well (R^2^ > 0.95, *p* < 0.01, shown in Figure 3a). From the relationship between *I* and the feed kaolin concentration in the MF process (shown in Figure 3b), *I* gradually increased from 2.58 × 10^10^ to 3.69 × 10^10^ to 4.80 × 10^10^ m^−2^ with feed kaolin concentrations from 10 to 50 to 100 mg/L, respectively. The linear fitting showed a very good linear positive correlation between *I* and the feed kaolin concentration (R^2^ > 0.99, *p* = 0.04), with the slope of 2 × 10^8^ m/g characterizing the cake layer specific resistance.

**Figure 4:**
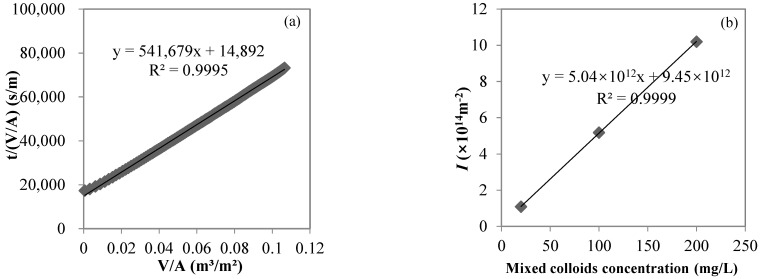
Cake layer model fitting for the UF process of a sodium alginate and bovine serum albumin (each 10 mg/L) mixed solution (**a**) and the correlation between *I* and the feed colloid concentration (**b**).

The DOC values of mixed sodium alginate and bovine serum albumin solutions with concentrations of 10, 50, and 100 mg/L were 5.21, 22.60, and 42.72 mg/L, respectively; the DOC values of UF permeate were 1.73, 4.68, and 12.36 mg/L, respectively, and the DOC rejection rates of UF were 66.8, 79.3, and 71.1%, respectively, indicating that the UF membrane with an MWCO of 100 kDa used in the study could effectively reject colloids, represented by sodium alginate and bovine serum albumin. The UF process closely followed the cake layer model in this study. Taking the filtration of a mixed 10 mg/L sodium alginate and 10 mg/L bovine serum albumin solution as an example, the cake layer model fit well (R^2^ > 0.99, *p* < 0.01, shown in Figure 4a). From the relationship between *I* and the feed colloid concentration in the UF process (shown in Figure 4b), *I* gradually increased from 1.08 × 10^14^ to 5.18 × 10^14^ to 1.02 × 10^15^ m^−2^ with feed colloid concentrations from 20 to 100 to 200 mg/L, respectively. The linear fitting showed a very good linear positive correlation between *I* and feed colloid concentrations (R^2^ > 0.99, *p* < 0.01), with the slope of 5 × 10^12^ m/g characterizing the cake (or gel) layer specific resistance.

**Figure 5:**
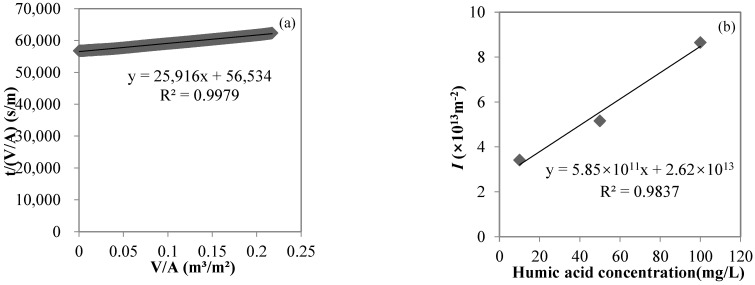
Cake layer model fitting for the NF process of a 10 mg/L humic acid solution (**a**) and the correlation between *I* and the influent humic acid concentration (**b**).

The UV_254_ values of the humic acid solutions with concentrations of 10, 50, and 100 mg/L were 0.332, 1.834, and 3.433 cm^−1^, respectively, while the UV_254_ values of the NF permeates were 0.025, 0.032, and 0.045 cm^−1^, respectively, and the UV_254_ rejection rates of NF were 92.5, 98.3, and 98.7%, respectively, indicating that the NF membrane with an MWCO of 300 Da used in the study could effectively reject dissolved organic matters, represented by humic acid. The NF process closely followed the cake layer model in this study. Taking the filtration of a 10 mg/L humic acid solution as an example, the cake layer model fit well (R^2^ > 0.99, *p* < 0.01, shown in Figure 5a). From the relationship between *I* and feed humic acid concentration in the NF process (shown in Figure 5b), *I* gradually increased from 3.42 × 10^13^ to 5.16 × 10^13^ to 8.65 × 10^13^ m^−2^, with feed humic acid concentrations from 10 to 50 to 100 mg/L, respectively. The linear fitting showed a very good linear positive correlation between *I* and the feed humic acid concentration (R^2^ > 0.98, *p* = 0.08), with the slope of 6 × 10^11^ m/g characterizing the cake (or gel) layer specific resistance.

The filtration processes of the MF, UF, and NF membranes feeding their respective targeted foulants (i.e., suspended solids, colloids, and dissolved organic matters) in this study closely followed the cake layer model, and the cake layer fouling index, *I*, showed a good linear positive correlation with the foulant concentration, demonstrating that *I* could serve as a quantitative index to characterize the membrane fouling potential, thus laying a theoretical foundation for the subsequent investigation of the gradient filtration of MF → UF → NF for wastewater samples. The linear fitting relationship between *I* and the feed foulant concentration showed that the specific resistance (5 × 10^12^ m/g) of the UF cake layer formed by colloids (i.e., sodium alginate and bovine serum albumin) per unit concentration > the specific resistance (6 × 10^11^ m/g) of the NF cake layer formed by dissolved organic matters (i.e., humic acid) per unit concentration >> the specific resistance (2 × 10^8^ m/g) of the MF cake layer formed by suspended solids (i.e., kaolin) per unit concentration.

### 3.2. Development of Cake Layer Fouling Potential Assessment Methodology in Gradient Filtration Based on SWW

#### 3.2.1. Rejection Performance during the Gradient Filtration of SWW

**Figure 6:**
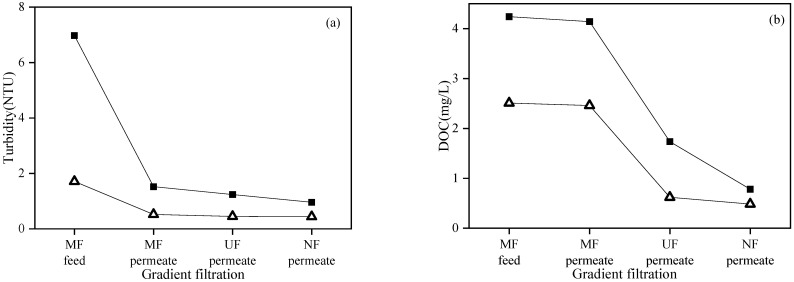

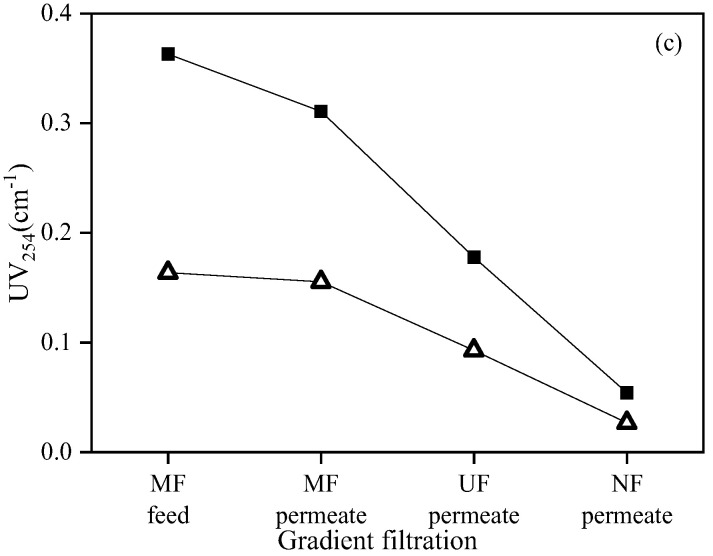
Turbidity (**a**), DOC (**b**), and UV_254_ (**c**) during gradient filtration of low- (△) and high-concentration (■) SWW.

Figure 6 shows the feed and permeate quality of each membrane in the gradient filtration of SWW containing high- and low-concentration model foulant mixtures. The turbidity, DOC, and UV_254_ of low- and high-concentration SWW (i.e., MF feed) were 1.71 and 6.98 NTU, 2.51 and 4.24 mg/L, 0.16 and 0.36 cm^−1^, respectively. For low- and high-concentration SWW, the turbidity rejection rates of MF, UF, and NF in gradient filtration were 69.8 and 78.2%, 13.5 and 18.6%, and 2.2 and 22.6%, respectively. The high turbidity rejection rate of MF verified its high rejection of the targeted foulants—suspended solids (i.e., kaolin). For low- and high-concentration SWW, the DOC rejection rates of MF, UF, and NF in gradient filtration were 1.9 and 2.4%, 74.9 and 58.1%, and 22.0 and 55.0%, respectively. The high rejection rate of DOC by UF verified its high rejection of the targeted foulants—colloids (i.e., sodium alginate and bovine serum albumin). For low- and high-concentration SWW, the UV_254_ rejection rates of MF, UF, and NF in gradient filtration were 5.1 and 14.4%, 40.3 and 42.8%, and 71.2 and 69.6%, respectively. The high UV_254_ rejection rate of NF verified its high rejection of the targeted foulants—dissolved organic matters (i.e., humic acid). It should be noted that turbidity, DOC, and UV_254_ were not solely specific to suspended solids, colloids, and dissolved organic matters, respectively, in this study. For example, sodium alginate and bovine serum albumin could generate turbidity, humic acid could generate DOC, and bovine serum albumin could generate UV_254_. This was why UF showed significant rejection on UV_254_ and NF showed significant rejection on DOC.

#### 3.2.2. Membrane Fouling Mechanism Analysis in Gradient Filtration Based on Single Models

**Figure 7:**
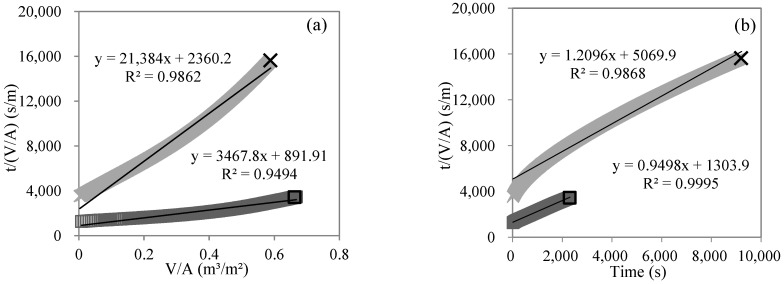

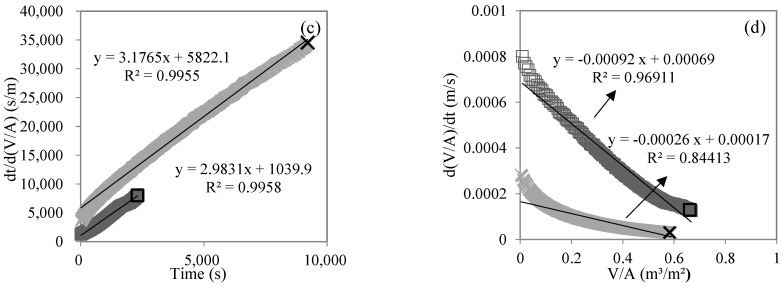
Linear fitting of single models, including cake layer (**a**), standard blocking (**b**), intermediate blocking (**c**), and complete blocking (**d**) for MF process during gradient filtration of low- (□) and high-concentration (×) SWW.

Figure 7 shows the fitting results of single models for the MF process in the gradient filtration of SWW. Benefitting from the high-frequency recording of permeate weight every 10 s in this study, the dense data points formed smooth curves for all subfigures. Based on the general standard of R^2^ > 0.95 employed in this study, the cake layer, standard blocking, and intermediate blocking models showed good linear fitting (R^2^ > 0.95, *p* < 0.01) for both low- and high-concentration SWW. The complete blocking model also showed good linear fitting (R^2^ = 0.9691, *p* < 0.01) for low-concentration SWW. If setting a very good linear fitting standard (e.g., R^2^ > 0.99), it was still achievable via selecting partial data (e.g., the final/initial third of data points for high/low-concentration SWW) to make good linear fittings for all four single models. The same methodology was employed for the UF and NF processes. Table 2 summarizes the linear fitting R^2^ of single models for the MF, UF, and NF processes in gradient filtration (*p* < 0.01). The simultaneous occurrence of two or more membrane fouling mechanisms not only existed in the MF process but also in the UF and NF processes. For example, during the NF process of low-concentration SWW, four single models (i.e., cake layer, standard blocking, intermediate blocking, and complete blocking) fit well, indicating the occurrence of statistical illusion in judging the fouling mechanism in the filtration process simply via single models [36,37,38].

#### 3.2.3. Membrane Fouling Mechanism Analysis in Gradient Filtration Based on Combined Models

**Figure 8:**
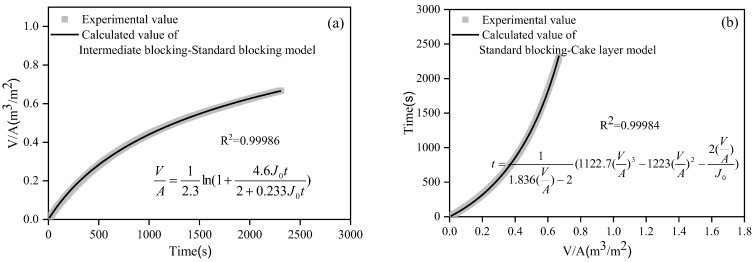
Nonlinear fitting of the combined models of intermediate blocking–standard blocking (**a**) and standard blocking–cake layer (**b**) for the MF process during the gradient filtration of low-concentration SWW.

Taking the MF process in the gradient filtration of low-concentration SWW as an example, Figure 8 shows the fitting results of the combined models of intermediate blocking–standard blocking and standard blocking–cake layer. The calculated values of the models were in very good agreement with the experimental values (R^2^ of 0.9998 and 0.9994, respectively, *p* < 0.01). Only one combined model fit well in the other membrane filtration processes (R^2^ > 0.98, *p* < 0.01). Table 3 summarizes the fitting results of the combined models in the gradient filtration processes (*p* < 0.01). For the MF process of the gradient filtration of low-concentration SWW, two combined models (i.e., intermediate blocking–standard blocking and standard blocking–cake layer) fit well, indicating the simultaneous occurrence of intermediate blocking, standard blocking, and cake layer fouling mechanisms. According to the characteristic parameters (*K_c_*, *K_i_*, *K_s_*, and *K_b_*) of nonlinear fitting, the contribution of each single fouling mechanism to flux reduction could be calculated [35,36]. In the combined model of intermediate blocking and standard blocking, the contributions of intermediate blocking and standard blocking were 91.3% and 8.7%, respectively, while in the combined model of standard blocking and cake layer, the contributions of standard blocking and cake layer were 64.1% and 35.1%, respectively. Therefore, three fouling mechanisms coexisted in the MF process of the gradient filtration of low-concentration SWW, and the cake layer was not the dominant fouling mechanism, which might be related to the interaction (e.g., noncovalent interaction and spatial effect) between suspended solids and colloids or dissolved organic matters in the SWW [14]. For the MF process of the gradient filtration of high-concentration SWW, only the combined model of complete blocking and cake layer fit well. The quantitative calculation showed that the contributions of complete blocking and cake layer were 88.2% and 11.8%, respectively, indicating that the cake layer was not the dominant fouling mechanism. For the UF and NF process of the gradient filtration of low- and high-concentration SWW, only the combined model of intermediate blocking and cake layer fit well. Because the characteristic parameter, *K_i_*, of the intermediate blocking model was almost zero, the quantitative calculation showed that the contribution of the cake layer was almost 100%, indicating that the cake layer was the absolute dominant fouling mechanism.

#### 3.2.4. Evaluation of Cake Layer Fouling Potential of SWW Based on Gradient Filtration

**Figure 9:**
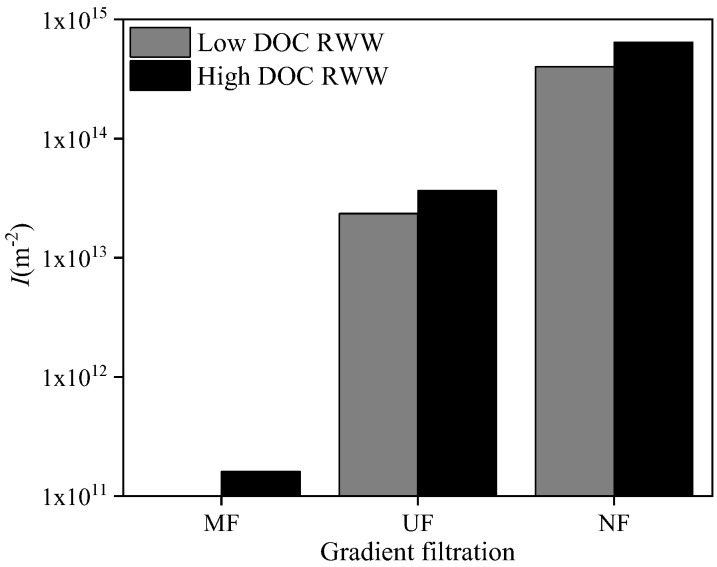
Cake layer fouling index, *I*, during gradient filtration of SWW.

Figure 9 shows the cake layer fouling index, *I*, of each membrane filtration process in the gradient filtration of SWW according to the above combined model fitting. The *I* values in the MF process of gradient filtration with low- and high-concentration SWW were 6.08 × 10^10^ and 1.44 × 10^12^ m^−2^, respectively, and the corresponding feed turbidity values of MF were 1.71 and 6.98 NTU, respectively. The *I* values of the UF process were 5.77 × 10^12^ and 1.02 × 10^13^ m^−2^, respectively, and the DOC values of UF feed (i.e., MF permeate) were 2.46 and 4.14 mg/L, respectively. The *I* values of the NF process were 1.40 × 10^14^ m^−2^ and 2.16 × 10^14^ m^−2^, respectively, and the UV_254_ values of NF feed (i.e., UF permeate) were 0.09 and 0.18 cm^−1^, respectively. The *I* of each membrane had a positive correlation with its targeted feed foulant concentration, indicating that the *I* values of the MF, UF, and NF processes could quantitatively evaluate the cake layer fouling potential of suspended solids, colloids, and dissolved organic matters, respectively. The order of *I* values in the different membrane filtration process was NF >> UF >> MF. Further considering the foulant concentration ratio in SWW (suspended solids/colloids/dissolved organic matter ratio of 4:1:3), it could be roughly estimated that the specific resistance order of the cake layer formed by foulants was NF > UF >> MF (i.e., dissolved organic matters > colloids >> suspended solids), similar to previous studies [32,33]. This was different from the order of specific resistance of the cake layer (i.e., UF > NF >> MF or colloids > dissolved organic matters >> suspended solids) in Section 3.1, where individual membranes and targeted foulants were applied. This could be related to the interactions of different foulants in SWW [14].

### 3.3. Cake Layer Fouling Potential Assessment via Gradient Filtration of RWW

#### 3.3.1. Rejection Performance in Gradient Filtration of RWW

**Figure 10:**
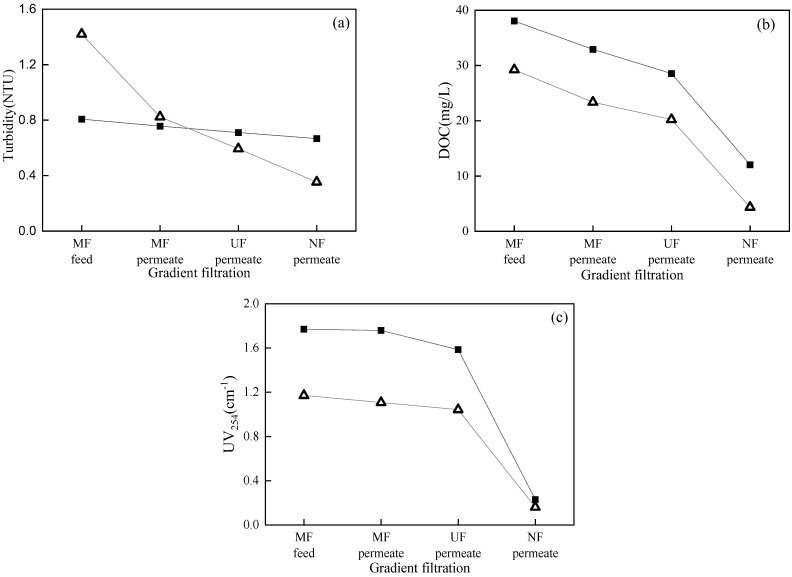
Turbidity (**a**), DOC (**b**), and UV_254_ (**c**) during gradient filtration of low- (△) and high-DOC (■) RWW.

Figure 10 shows the feed and permeate qualities of each membrane in the gradient filtration of RWW (i.e., the supernatant of MBR treating dyeing and finishing wastewater) with two different DOC concentrations sampled in different seasons. The DOC, turbidity, and UV_254_ values of low- and high-DOC RWW were 29.22 and 38.03 mg/L, 1.42 and 0.81 NTU, and 1.17 and 1.77 cm^−1^, respectively. The higher turbidity in low-DOC than high-DOC RWW was mainly due to the worse sludge settleability when sampling MBR sludge. The turbidity rejection rates of low- and high-DOC RWW by MF, UF, and NF were 42.0% and 6.2%, 27.9% and 6.2%, and 40.4% and 6.1%, respectively, indicating that higher turbidity in RWW could result in higher rejection by membranes. The DOC rejection rates of low- and high-DOC RWW by MF, UF, and NF were 20.1% and 13.4%, 13.4% and 13.3%, and 78.5% and 57.8%, respectively. The DOC rejection order of NF >> MF > UF might be related to the bimodal distribution of organics in the MBR supernatant [39,40], where the minor submicron biopolymers could be rejected directly by MF and the major low-molecular-weight organics less than 1 kDa could only be rejected by NF, thus resulting in low rejection by UF in this study. The UV_254_ rejection rates of low- and high-DOC RWW by MF, UF, and NF were 5.5% and 0.6%, 5.8% and 9.9%, and 84.5% and 85.5%, respectively. The high rejection of both DOC and UV_254_ by NF indicated that low-molecular-weight organic compounds with benzene rings and unsaturated bonds (e.g., humic and fulvic substances) could be the major components of RWW in this study.

#### 3.3.2. Membrane Fouling Mechanism Analysis and Cake Layer Fouling Potential Evaluation in Gradient Filtration of RWW

Table 4 shows the linear fitting results of single models for the gradient filtration of RWW. Similar to SWW, the linear fitting of single models for the gradient filtration of RWW showed the simultaneous occurrence of multiple fouling mechanisms caused by the statistical illusion, with the only exception being the NF process of low-DOC RWW, where none of the single models fit well.

Table 5 presents the nonlinear fitting results of the combined models for RWW. For the MF process of low-DOC RWW, only the combined model of intermediate blocking and standard blocking fit well. The contribution of intermediate blocking was calculated as 84.7%, indicating the dominant intermediate blocking fouling in the MF process that neither the standard blocking fitted by single models nor the cake layer generally considered, which might be due to very low concentration of suspended solids in RWW [23]. For the UF process of low-DOC RWW, only the combined model of intermediate blocking and cake layer fit well. Although the fitting characteristic parameter, *K_i_*, of the intermediate blocking model did not pass the *t* test, the contribution of intermediate blocking could be neglected, considering the very small order of magnitude of *K_i_*, resulting in the dominant cake layer fouling in the UF process. For the NF process of low-DOC RWW, only the combined model of intermediate blocking and cake layer fit well, where the cake layer fouling dominated due to the same reason as in the UF process. For the MF process of high-DOC RWW, both the combined model of intermediate blocking and cake layer and the combined model of standard blocking and cake layer fit well. The contributions of the cake layer were calculated as 97.6% and 77.1% in the combined model of intermediate blocking and cake layer and the combined model of standard blocking and cake layer, respectively, resulting in the dominant cake layer fouling in the MF process. For the UF process of high-DOC RWW, only the combined model of intermediate blocking and cake layer fit well. Cake layer fouling dominated in the UF process based on the very small value (almost zero) of *K_i_*. For the NF process of high-DOC RWW, only the combined model of intermediate blocking and cake layer fit well. The contribution of the cake layer was calculated as 57.8%, indicating major cake layer fouling in the NF process.

**Figure 11:**
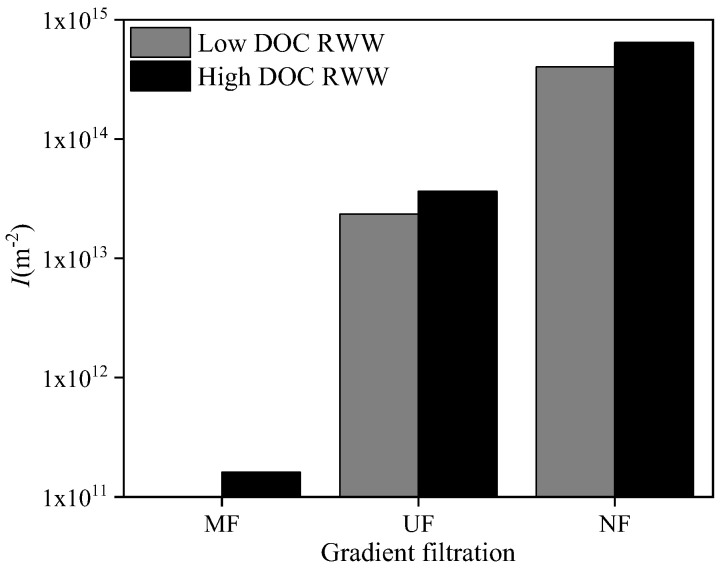
Cake layer fouling index, *I*, during gradient filtration of RWW.

Based on the above-mentioned fouling mechanism analysis, the cake layer fouling index, *I*, was calculated during the gradient filtration of RWW (shown in Figure 11), except for the MF process of low-DOC RWW with dominant pore blocking fouling. For low- and high-DOC RWW, the *I* values of the UF process were 2.35 × 10^13^ and 3.65 × 10^13^ m^−2^, corresponding to the UF rejected DOC values of 3.14 and 4.38 mg/L, respectively. For low- and high-DOC RWW, the *I* values of the NF process were 4.02 × 10^14^ and 6.44 × 10^14^ m^−2^, corresponding to the NF rejected DOC values of 15.86 and 16.51 mg/L, respectively. Similar to SWW, the *I* values of the UF and NF processes showed a positive correlation with foulant concentration, indicating their capability to characterize the fouling potential of RWW quantitatively. A further comparison between *I* and the rejected DOC of the UF and NF processes for both low- and high-DOC RWW showed that the fouling layer formed on the NF membrane by dissolved organic matters had a significantly higher specific resistance than that formed on the UF membrane by colloids. For high-DOC RWW, the *I* of the MF process was 1.6 × 10^11^ m^−2^, and the MF rejected DOC was 5.1 mg/L. A further comparison between *I* and the rejected DOC values of the MF and UF processes for high-DOC RWW showed that the fouling layer formed on the MF membrane by dissolved organic matters had a far lower specific resistance than that formed on the UF membrane by colloids. These results were also in agreement with the above-mentioned SWW.

## 4. Conclusions

The gradient filtration of MF (0.45 μm) → UF (100 kDa) → NF (300 Da) with combined model correlation and the calculation of the cake layer fouling index, *I*, was developed to quantitatively characterize the feed fouling potential for RO fouling prevention and control in this study. The following conclusions could be drawn:

The rejection rates of MF, UF, and NF for individual targeted model foulants (i.e., kaolin representing suspended solids, sodium alginate and bovine serum albumin representing colloids, and humic acid representing dissolved organic matters) were 79.6%, 72.4%, and 96.5%, respectively, during constant-pressure filtration tests where the cake layer was the dominant membrane fouling mechanism and the cake layer fouling index, *I*, showed a positive linear correlation with feed foulant concentration. In the MF → UF → NF gradient filtration of low- and high-concentration SWW (i.e., model foulant mixture), the rejection rates of MF, UF, and NF for turbidity, DOC, and UV_254_ were 69.8% and 78.2%, 74.9% and 58.1%, and 71.2% and 69.6%, respectively. A single-model analysis showed that two or more models fit well for each membrane filtration process due to statistical illusion. A combined-model analysis confirmed the occurrence of multiple fouling mechanisms, and the contribution of each fouling mechanism could be further quantified. In the MF process of SWW, the cake layer was not the dominant fouling mechanism due to the interaction between suspended solids and colloids or dissolved organic matters. In the UF and NF processes of SWW, the cake layer was the dominant fouling mechanism. In MF → UF → NF gradient filtration, the *I* values of each membrane were positively correlated with the corresponding targeted foulant concentrations (suspended solids, colloids, and dissolved organic matters). The orders of *I* and the cake layer specific resistance were NF >> UF >> MF and NF > UF >> MF. Thus, the gradient filtration method was preliminarily established to quantitatively characterize the cake layer fouling potential of different foulants.

RWW (i.e., the sludge supernatant of MBR for dyeing and finishing wastewater treatment) was employed to verify the applicability of the proposed MF → UF → NF gradient filtration method. For low- and high-DOC RWW, the rejection rates of MF, UF, and NF for turbidity were 42.0% and 6.2%, 27.9% and 6.2%, and 40.4% and 6.1%, respectively; the rejection rates of MF, UF, and NF for DOC were 20.1% and 13.4%, 13.4% and 13.3%, and 78.5% and 57.8%, respectively; and the rejection rates of MF, UF, and NF for UV_254_ were 5.5% and 0.6%, 5.8% and 9.9%, and 84.5% and 85.5%, respectively. Similar to SWW, single models failed to identify fouling mechanisms, but combined models successfully performed this task. In the MF process of low-DOC RWW, the cake layer was not formed, possibly due to the very low concentration of suspended solids. Similar to SWW, the *I* values of the UF and NF processes showed a positive correlation with foulant concentration. The order of *I* was NF >> UF >> MF, and the order of cake layer specific resistance was NF > UF >> MF. Therefore, the proposed MF → UF → NF gradient filtration would be promising for quantitative cake layer fouling potential evaluation.

## Figures and Tables

**Table 1:** Membrane fouling models for constant-pressure filtration.

Model Name	Equation *	Characteristic Parameters	Schematic Diagram
Cake layer	$\frac{t}{\left(V/A\right)}=\frac{1}{J_{0}}+\frac{K_{c}}{2}\left(V/A\right)$	*K_c_* (s/m^2^)	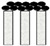
Complete blocking	$\frac{d\left(V/A\right)}{dt}=J_{0}-K_{b}\left(V/A\right)$	*K_b_* (s^−1^)	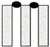
Intermediate blocking	$\frac{dt}{d\left(V/A\right)}=\frac{1}{J_{0}}+K_{i}t$	*K_i_* (m^−1^)	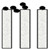
Standard blocking	$\frac{t}{\left(V/A\right)}=\frac{1}{J_{0}}+\frac{K_{s}}{2}t$	*K_s_* (m^−1^)	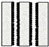
Complete blocking–Cake layer	$\frac{V}{A}=\frac{J_{0}}{K_{b}}\left(1-\exp\left(\frac{-K_{b}}{K_{c}J_{0}^{2}}\left(\sqrt{1+2K_{c}J_{0}^{2}t}-1\right)\right)\right)$	*K_c_* (s/m^2^), *K_b_* (s^−1^)	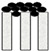
Intermediate blocking–Cake layer	$\frac{V}{A}=\frac{1}{K_{i}}\ln\left(1+\frac{K_{i}}{K_{c}J_{0}}\left(\sqrt{1+2K_{c}J_{0}^{2}t}-1\right)\right)$	*K_c_* (s/m^2^), *K_i_* (m^−1^)	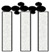
Complete blocking–Standard blocking	$\frac{V}{A}=\frac{J_{0}}{K_{b}}\left(1-\exp\left(\frac{-2K_{b}t}{2+K_{s}J_{0}t}\right)\right)$	*K_b_* (s^−1^), *K_s_* (m^−1^)	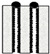
Intermediate blocking–Standard blocking	$\frac{V}{A}=\frac{1}{K_{i}}\ln\left(1+\frac{2K_{i}J_{0}t}{2+K_{s}J_{0}t}\right)$	*K_i_* (m^−1^), *K_s_* (m^−1^)	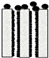
Standard blocking–Cake layer	$t=\frac{1}{K_{s}\left(\frac{V}{A}\right)-2}\left(\frac{K_{s}K_{c}{\left(\frac{V}{A}\right)}^{3}}{2}-K_{c}{\left(\frac{V}{A}\right)}^{2}-\frac{2\left(\frac{V}{A}\right)}{J_{0}}\right)$	*K_c_* (s/m^2^), *K_s_* (m^−1^)	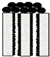

*: *t* is filtration time (s), *V* is permeate volume (m^3^), *A* is membrane area (m^2^), *J*_0_ is initial membrane flux (m^3^/m^2^·s).

**Table 2:** R^2^ of single model fitting for gradient filtration of SWW.

	Cake Layer		Standard Blocking		Intermediate Blocking		Complete Blocking	
Membrane	Low-Concentration SWW	High-Concentration SWW	Low-Concentration SWW	High-Concentration SWW	Low-Concentration SWW	High-Concentration SWW	Low-Concentration SWW	High-Concentration SWW
MF	0.9494	0.9862	0.9995	0.9868	0.9958	0.9955	0.9691	0.8441
UF	0.9996	0.9771	0.9704	0.9419	0.9783	0.9739	0.8213	0.8116
NF	0.9529	0.8367	0.9776	0.8242	0.9786	0.7607	0.9698	0.8024

**Table 3:** Fitting results of combined models during gradient filtration of SWW.

Concentration	Gradient Filtration	Combined Model	Parameter Value	Nonlinear Fitting R^2^
low-concentration SWW	MF	Intermediate blocking–Standard blocking	*K_i_* = 2.30 m^−1^ *K_s_* = 0.223 m^−1^	0.99986
		Standard blocking–Cake layer	*K_s_* = 1.836 m^−1^ *K_c_* = 1223 s/m^2^	0.99984
	UF	Intermediate blocking–Cake layer	*K_i_* = 2.61 × 10^−7^ m^−1^	0.9994
			*K_c_* = 5.8 × 10^4^ s/m^2^	
	NF	Intermediate blocking–Cake layer	*K_i_* = 4.03 × 10^−5^ m^−1^	0.9988
			*K_c_* = 1.41 × 10^5^ s/m^2^	
high-concentration SWW	MF	Complete blocking–Cake layer	*K_b_* = 0.0178 s^−1^	0.9946
			*K_c_* = 2.89 × 10^4^ s/m^2^	
	UF	Intermediate blocking–Cake layer	*K_i_* = 1.74 × 10^−7^ m^−1^	0.9893
			*K*_c_ = 1.03 × 10^5^ s/m^2^	
	NF	Intermediate blocking–Cake layer	*K_i_* = 1.85 × 10^−11^ m^−1^	0.995
			*K_c_* = 2.17 × 10^5^ s/m^2^	

**Table 4:** R^2^ of single model fitting for gradient filtration of RWW.

	Cake Layer		Standard Blocking		Intermediate Blocking		Complete Blocking	
Membrane	Low-DOC RWW	High-DOC RWW	Low-DOC RWW	High-DOC RWW	Low-DOC RWW	High-DOC RWW	Low-DOC RWW	High-DOC RWW
MF	---	0.9986	0.9996	0.9934	0.9823	0.9913	0.9117	0.9644
UF	0.9748	0.9913	0.9518	0.9851	0.6943	0.804	0.8857	0.9207
NF	0.8930	0.968	0.8809	09811	0.8554	0.953	0.8824	0.9681

**Table 5:** Fitting results of combined models during gradient filtration of RWW.

Type	Gradient Filtration	Combined Model	Parameter Value	Nonlinear Fitting R^2^
Low-DOC RWW	MF	Intermediate blocking–Standard blocking	*K_i_* = 3.88 m^−1^, *K_s_* = 0.70 m^−1^	0.9988
	UF	Intermediate blocking–Cake layer	*K_i_* = 9.05 × 10^−10^ m^−1^ *	0.9914
			*K_c_* = 2.36 × 10^5^ s/m^2^	
	NF	Intermediate blocking–Cake layer	*K_i_* = 2.36 × 10^−6^ m^−1^ *	0.9913
			*K_c_* = 4.04 × 10^5^ s/m^2^	
High-DOC RWW	MF	Intermediate blocking–Cake layer	*K_i_* = 0.0516 m^−1^, *K_c_* = 3226 s/m^2^	0.99997
		Standard blocking–Cake layer	*K_s_* = 0.468 m^−1^, *K_c_* = 2532 s/m^2^	0.99996
	UF	Intermediate blocking–Cake layer	*K_i_* = 2.24 × 10^−11^ m^−1^	0.9978
			*K_c_* = 3.67 × 10^5^ s/m^2^	
	NF	Standard blocking–Cake layer	*K_s_* = 5.97 m^−1^	0.9999
			*K_c_* = 6.47 × 10^5^ s/m^2^	

*: failed the *t* test at the significance level of 0.05.

## Data Availability

Not applicable.

## References

[1] Biesheuvel P.M., Porada S., Elimelech M., Dykstra J.E. (2022). Tutorial review of reverse osmosis and electrodialysis. J. Membr. Sci..

[2] Moossa B., Trivedi P., Saleem H., Zaidi S.J. (2022). Desalination in the GCC countries- a review. J. Clean. Prod..

[3] Park J., Lee S. (2022). Desalination Technology in South Korea: A Comprehensive Review of Technology Trends and Future Outlook. Membranes.

[4] Ruan G.L., Wang M., An Z.H., Xu G.R., Ge Y.H., Zhao H.L. (2021). Progress and Perspectives of Desalination in China. Membranes.

[5] Lin S.S., Zhao H.Y., Zhu L.P., He T., Chen S.F., Gao C.J., Zhang L. (2020). Seawater desalination technology and engineering in China: A review. Desalination.

[6] Zhang Z.W., Wu Y.H., Luo L.W., Li G.L., Li Y.B., Hu H.Y. (2021). Application of disk tube reverse osmosis in wastewater treatment: A review. Sci. Total Environ..

[7] Jeffrey P., Yang Z., Judd S.J. (2022). The status of potable water reuse implementation. Water Res..

[8] Yang J.Q., Monnot M., Ercolei L., Moulin P. (2020). Membrane-Based Processes Used in Municipal Wastewater Treatment for Water Reuse: State-of-the-Art and Performance Analysis. Membranes.

[9] Jafari M., Vanoppen M., van Agtmaal J.M.C., Cornelissen E.R., Vrouwenvelder J.S., Verliefde A., van Loosdrecht M.C.M., Picioreanu C. (2020). Cost of fouling in full-scale reverse osmosis and nanofiltration installations in the Netherlands. Desalination.

[10] Jiang S.X., Li Y.N., Ladewig B.P. (2017). A review of reverse osmosis membrane fouling and control strategies. Sci. Total Environ..

[11] Matin A., Laoui T., Falath W., Farooque M. (2020). Fouling control in reverse osmosis for water desalination & reuse: Current practices & emerging environment-friendly technologies. Sci. Total Environ..

[12] Qrenawi L.I., Abuhabib A.A. (2020). A review on sources, types, mechanisms, characteristics, impacts and control strategies of fouling in RO membrane systems. Water Treat..

[13] AlSawaftah N., Abuwatfa W., Darwish N., Husseini G. (2021). A Comprehensive Review on Membrane Fouling: Mathematical Modelling, Prediction, Diagnosis, and Mitigation. Water.

[14] Xu H., Xiao K., Wang X.M., Liang S., Wei C.H., Wen X.H., Huang X. (2020). Outlining the Roles of Membrane-Foulant and Foulant-Foulant Interactions in Organic Fouling During Microfiltration and Ultrafiltration: A Mini-Review. Front. Chem..

[15] Badruzzaman M., Voutchkov N., Weinrich L., Jacangelo J.G. (2019). Selection of pretreatment technologies for seawater reverse osmosis plants: A review. Desalination.

[16] Abushaban A., Salinas-Rodriguez S.G., Pastorelli D., Schippers J.C., Mondal S., Goueli S., Kennedy M.D. (2021). Assessing Pretreatment Effectiveness for Particulate, Organic and Biological Fouling in a Full-Scale SWRO Desalination Plant. Membranes.

[17] Sim L.N., Chong T.H., Taheri A.H., Sim S.T.V., Lai L., Krantz W.B., Fane A.G. (2018). A review of fouling indices and monitoring techniques for reverse osmosis. Desalination.

[18] Jin Y., Lee H., Park C., Hong S. (2020). ASTM Standard Modified Fouling Index for Seawater Reverse Osmosis Desalination Process: Status, Limitations, and Perspectives. Sep. Purif. Rev..

[19] Abushaban A., Salinas-Rodriguez S.G., Philibert M., Le Bouille L., Necibi M.C., Chehbouni A. (2022). Biofouling potential indicators to assess pretreatment and mitigate biofouling in SWRO membranes: A short review. Desalination.

[20] (2014). Standard Test Method for Silt Density Index (SDI) of Water.

[21] Alhadidi A., Blankert B., Kemperman A.J.B., Schurer R., Schippers J.C., Wessling M., van der Meer W.G.J. (2013). Limitations, improvements and alternatives of the silt density index. Desalin Water Treat..

[22] Yoo H.Y., Lee Y.S., Oh H.K., Kim J.O. (2021). Silt density index as a fouling propensity parameter of various membrane materials using dissolved organic matter. J. Water Process Eng..

[23] Wei C.H., Laborie S., Ben Aim R., Amy G. (2012). Full utilization of silt density index (SDI) measurements for seawater pre-treatment. J. Membr. Sci..

[24] (2015). Standard Test Method for Modified Fouling Index (MFI-0.45) of Water.

[25] Jin Y., Ju Y., Lee H., Hong S. (2015). Fouling potential evaluation by cake fouling index: Theoretical development, measurements, and its implications for fouling mechanisms. J. Membr. Sci..

[26] Boerlage S.F.E., Kennedy M., Aniye M.P., Schippers J.C. (2003). Applications of the MFI-UF to measure and predict particulate fouling in RO systems. J. Membr. Sci..

[27] Khirani S., Ben Aim R., Manero M.H. (2006). Improving the measurement of the Modified Fouling Index using nanofiltration membranes (NF-MFI). Desalination.

[28] Boerlage S.F.E., Kennedy M.D., Dickson M.R., El-Hodali D.E.Y., Schippers J.C. (2002). The modified fouling index using ultrafiltration membranes (MFI-UF): Characterisation, filtration mechanisms and proposed reference membrane. J. Membr. Sci..

[29] Salinas-Rodriguez S.G., Amy G.L., Schippers J.C., Kennedy M.D. (2015). The Modified Fouling Index Ultrafiltration constant flux for assessing particulate/colloidal fouling of RO systems. Desalination.

[30] Sim L.N., Ye Y., Chen V., Fane A.G. (2010). Crossflow Sampler Modified Fouling Index Ultrafiltration (CFS-MFIUF)-An alternative Fouling Index. J. Membr. Sci..

[31] Ju Y., Hong S. (2014). Nano-colloidal fouling mechanisms in seawater reverse osmosis process evaluated by cake resistance simulator-modified fouling index nanofiltration. Desalination.

[32] Yu Y., Lee S., Hong K., Hong S. (2010). Evaluation of membrane fouling potential by multiple membrane array system (MMAS): Measurements and applications. J. Membr. Sci..

[33] Ju Y., Hong I., Hong S. (2015). Multiple MFI measurements for the evaluation of organic fouling in SWRO desalination. Desalination.

[34] Xu P., Drewes J.E., Kim T.U., Bellona C., Amy G. (2006). Effect of membrane fouling on transport of organic contaminants in NF/RO membrane applications. J. Membr. Sci..

[35] Bolton G., LaCasse D., Kuriyel R. (2006). Combined models of membrane fouling: Development and application to microfiltration and ultrafiltration of biological fluids. J. Membr. Sci..

[36] Huang B., Gu H.K., Xiao K., Qu F.S., Yu H.R., Wei C.H. (2020). Fouling Mechanisms Analysis via Combined Fouling Models for Surface Water Ultrafiltration Process. Membranes.

[37] Li R.H., Gao B.Y., Wang W.Y., Yue Q.Y., Wang Y. (2019). Floc properties and membrane fouling in coagulation/ultrafiltration process for the treatment of Xiaoqing River: The role of polymeric aluminum-polymer dual-coagulants. Chemosphere.

[38] Xing J.J., Liang H., Xu S.Q., Chuah C.J., Luo X.S., Wang T.Y., Wang J.L., Li G.B., Snyder S.A. (2019). Organic matter removal and membrane fouling mitigation during algae-rich surface water treatment by powdered activated carbon adsorption pretreatment: Enhanced by UV and UV/chlorine oxidation. Water Res..

[39] Sun J.Y., Xiao K., Mo Y.H., Liang P., Shen Y.X., Zhu N.W., Huang X. (2014). Seasonal characteristics of supernatant organics and its effect on membrane fouling in a full-scale membrane bioreactor. J. Membr. Sci..

[40] Wei C.H., Amy G. (2013). Sludge Water Characteristics Under Different Separation Methods from a Membrane Bioreactor. Sep. Sci. Technol..

